# Supplementary material for: Properties of cell signaling pathways and gene expression systems operating far from steady-state
Source: Sci Rep. 2018 Nov 19;8:17035. doi: 10.1038/s41598-018-34766-0 (PMC6242903; doi:10.1038/s41598-018-34766-0)
Supplement: Supplementary file 1 — Supplementary Information [file 41598_2018_34766_MOESM1_ESM.pdf]

Supplementary Table

Parameter	Interval for simulation	Interval from Huang and Ferrell [1]	Interval from Bhalla and Iyengar [2]
$\mathrm{k_1}, \mathrm{k_2}$	[6.3, 600] min^{-1}	[150, 150] min^{-1}	[6.3, 600] min^{-1}
$\mathrm{a_1}, \mathrm{a_2}$	[18.018, 2500] $nM\ \mathrm{min}^{-1}$	[2500, 2500] $nM\ \mathrm{min}^{-1}$	[18.018, 4545.45] $nM\ \mathrm{min}^{-1}$
$\mathrm{d_1}, \mathrm{d_2}$	[25.2, 2400] min^{-1}	[600, 600] min^{-1}	[25.2, 2400] min^{-1}
$\mathrm{E_T}$	[0.3, 224]nM	[0.3, 120] nM	[3.2, 224] nM
$\mathrm{S_T}$	[3, 1000] nM	[3, 1200] nM	[100, 100] nM

Table S1. For each of the parameters of the cycle, we indicate the interval considered for simulation and the intervals given in the paper by Huang and Ferrel and in that by Bhalla and Iyengar. This criterion for sampling intervals for a CM cycle was taken from [4].

Supplementary Information

Properties of cell signaling pathways and gene expression systems operating far from steady-state

Juan Pablo Di-Bella[1,2], Alejandro Colman-Lerner[1,2]* and Alejandra C. Ventura[1,2]*

[1] Department of Physiology, Molecular and Cellular Biology, University of Buenos Aires, Buenos Aires, Argentina

[2] Institute of Physiology, Molecular Biology and Neurosciences, National Research Council (CONICET), Buenos Aires, Argentina

Correspondence Tel: +54 11 4576 3368; E-mail: colman-lerner@fbmc.fcen.uba.ar and alejvent@fbmc.fcen.uba.ar

* equally contributing authors

1. Ligand-receptor system

The simplest binding model is a receptor R that binds a ligand L forming a complex C, and is described by the following reaction:

$$R + L(t) \underset{k_{off}}{\overset{k_{on}}{\rightleftarrows}} C \qquad \text{(S1)}$$

Using mass action kinetics and the conservation relation $R_{TOT} = R + C$, the dynamics of the complex C is described by the following differential equation:

$$\frac{dC}{dt} = k_{on}L(t)(R_{TOT} - C) - k_{off}C\ , \qquad \text{(S2)}$$

where R was obtained from the conservation relation, $R = R_{TOT} - C$, to reduce the number of variables. We introduced the following definitions: $x(t_n) = L(t_n)/K_D$ is the amount of ligand relative to the dissociation constant for the binding-unbinding reaction ($K_D = k_{off}/k_{on}$), $y = C/R_T$ is the amount of ligand-receptor complex relative to the total amount of receptors, and $t_n = t/t_{ref}$ is the time expressed in units of a reference time $t_{ref} = 1/k_{off}$. x, y, and t_n are dimensionless variables and transform equation (S2) into:

$$\frac{dy}{dt_n} = x(t_n)(1 - y) - y \qquad \text{(S3)}$$

We further assumed that the amount of ligand increases following an exponential function characterized by a maximum value L_{max} and a characteristic time τ_L, resulting in $x_{max} = L_{max}/K_d$ and $\tau_{Ln} = \tau_L/t_{ref}$, which connects the time-scale associated with the ligand accumulation (τ_L) with that of the binding-unbinding process ($t_{ref} = 1/k_{off}$):

$$x(t_n) = x_{max}\,(1 - exp(-t_n\,/\tau_{Ln})) \qquad \text{(S4)}$$

We here consider two limit cases: fast stimulation with slow binding/unbinding and slow stimulation with fast binding/unbinding.

1a. Ligand-receptor system: fast ligand accumulation and slow binding/unbinding ($\tau_{Ln} \ll 1$)

For $\tau_{Ln} \ll 1$, equation S4 is reduced to $x(t_n) \cong x_{max}$, so this limit case resembles the system we described in [3] and in the Introduction of this paper, *i.e*, a receptor *R* interacting with a step-like temporal profile of ligand *L*. The solution of equation S2 is, then:

$$y(t_n, x) = \frac{x}{1+x}\left(1 - e^{(-t_n(1+x))}\right) \qquad \text{(S5)}$$

The steady-state of equation S5, which is obtained by taking the limit $t_n \to \infty$, has an $n_H = 1$ and $EC_{50} = 1$. We are interested in obtaining the EC_{50} and n_H for y versus x, for any given time, which will result in two relationships: $EC_{50}(t_n)$ and $n_H(t_n)$.

To obtain these functions we need to solve the same equation with different values. Hence, we define a real number ϵ as the corresponding fraction: $\epsilon = 0.5$ for the EC_{50}, $\epsilon = 0.1$ for the EC_{10} and $\epsilon = 0.9$ for the EC_{90}. Then,

$$y(t_n, x_\epsilon) = \frac{x_\epsilon}{1+x_\epsilon}\left[1 - \exp\left(-t_n(1 + x_\epsilon)\right)\right] = \epsilon, \qquad \text{(S6)}$$

where x_ϵ is the value of the input that gives ϵ as the output (e.g. for $\epsilon = 0.5$, $x_\epsilon = EC_{50}$). From equation S6 we can easily compute the time as a function of the remaining variables (i.e. the inverse function)

$$t_n = -\frac{1}{1+x_\epsilon}\log\left(\frac{x_\epsilon(1-\epsilon\,)-\epsilon}{x_\epsilon}\right) \qquad \text{(S7)}$$

Based on this last expression, EC_{50} versus time results in the plot we show in Fig. 1D in the main text. According to this plot, EC_{50} decreases when time increases, reaching a value of 1 at binding equilibrium.

The Hill coefficient is defined as

$$n_H = log10(81)/log10(EC_{90}/EC_{10}) \qquad \text{(S8)}$$

We obtained an approximated $n_H = 1.4$ in the limit of low values of t_n. This value is achieved by deriving $t_n(EC_{90})$ and $t_n(EC_{10})$ in the same way we derived $t_n(EC_{50})$, and neglecting terms as follows:

$$t_n = \frac{-1}{(1+EC_{10})}\log\left(\frac{EC_{10}0.9-0.1}{EC_{10}}\right) \sim \frac{-1}{(1+EC_{10})}\log(0.9) \qquad \text{(S9.a)}$$

$$t_n = \frac{-1}{(1+EC_{90})}\log\left(\frac{EC_{90}0.1-0.9}{EC_{90}}\right) \sim \frac{-1}{(1+EC_{90})}\log(0.1) \qquad \text{(S9.b)}$$

These approximations are valid because at short times ($t_n << 1$), both EC_{90} and EC_{10} are much greater than one, similar to the behavior of EC_{50} (Figure 1D, main text). From the last two formulas we obtained the ratio EC_{90}/EC_{10}:

$$\frac{EC_{90}}{EC_{10}} = \frac{-\log(0.1)-t_n}{-\log(0.9)-t_n} \qquad \text{(S10)}$$

Which results in 21.85 when t_n is neglected, using equation (S8) this leads to $n_H = 1.42$. This approximated result indicates that the sensitivity goes from about 1.4, in the limit of low values of t_n to 1, in the limit of $t_n \to \infty$, and it means that before reaching equilibrium the sensitivity is higher than when equilibrium is reached.

1b. Ligand-receptor system: slow ligand accumulation with fast binding/unbinding

It is convenient, in this section, to re-define the dimensionless time so that it is expressed relative to the slowest time-scale in the system (τ_L, ligand accumulation time). In this way: $t^{\sim} = t/\tau_L$, resulting in

$$\kappa\frac{dy}{dt^{\sim}} = x(t^{\sim})(1-y) - y \qquad \text{(S11)}$$

where, as before, $x(t^{\sim}) = L(t^{\sim})/K_D$ is the normalized input and $y = C/R_T$ is the normalized output. $\kappa = 1/(k_{off}\tau_L)$ is a dimensionless parameter that is a ratio of the time characterizing the binding-unbinding reaction and the time characterizing ligand accumulation. We consider the limit of slow ligand accumulation with fast binding/unbinding, thus, $\kappa \ll 1$. In this limit, the (binding) reaction is fast, equilibrating rapidly and remaining in near-equilibrium even as the variable x slowly changes. Thus, we take the quasi-steady-state approximation $\kappa\, dy/dt^{\sim} = 0$, resulting in:

$$y(t^{\sim}, x(t^{\sim})) = \frac{x(t^{\sim})}{1+x(t^{\sim})} \qquad \text{(S12)}$$

The steady-state of the input-output curve is, as before, $x/(1+x)$, a hyperbolic curve characterized by $EC_{50} = 1$ and dynamic range $EC_{90}/EC_{10} = 81$. However, if we consider the time-dependent input-output curve, $i.e.$, y versus x for a given time $t^{\sim}$, we find a time-dependent EC_{50}:

$$EC_{50} = \frac{1}{1-exp(-t^{\sim})} \qquad \text{(S13)}$$

Interestingly, in this case the dynamic range is 81 (and thus $n_H = 1$) for every value of $t^{\sim}$.

2. Covalent modification cycles

1a. Mechanistic description and parameter values

A covalent modification cycle (CM) may be described by the following reactions:

$$E_a + S \underset{d_1}{\overset{a_1}{\rightleftharpoons}} E_a S \overset{k_1}{\rightharpoonup} S^* + E_a \qquad \text{(S14.a)}$$

$$E_d + S^* \underset{d_2}{\overset{a_2}{\rightleftharpoons}} E_d S^* \overset{k_2}{\rightharpoonup} S + E_d \qquad \text{(S14.b)}$$

where E_a is the kinase, E_d is the phosphatase, S the unmodified substrate, S* the modified substrate, E_aS the complex between S and the enzyme that modifies the substrate (for example, if the enzyme were a kinase, by adding a phosphate group), and E_dS^* the complex between S* and the enzyme that removes the modification (for example a phosphatase). a_i are the association rates, d_i the dissociation rates, and k_i the catalytic rates ($i = 1$ for the forward reaction and $i = 2$ for the reverse reaction).

Applying the law of mass action, the kinetic equations governing the time evolution of such a system are:

$$\frac{dE_a}{dt} = -a_1 \cdot S \cdot E_a + (d_1 + k_1) \cdot E_aS \qquad \text{(S15.a)}$$

$$\frac{dS}{dt} = -a_1 \cdot S \cdot E_a + k_2 \cdot E_dS^* + d_1 \cdot E_aS \qquad \text{(S15.b)}$$

$$\frac{dS^*}{dt} = k_1 \cdot IS + d_2 \cdot E_dS^* - a_2 \cdot E_d \cdot S^* \qquad \text{(S15.c)}$$

$$\frac{dE_d}{dt} = -a_2 \cdot S^* \cdot E_d + (d_2 + k_2) \cdot E_dS^* \qquad \text{(S15.d)}$$

$$\frac{dE_aS}{dt} = a_1 \cdot E_a \cdot S - (d_1 + k_1) \cdot E_aS \qquad \text{(S15.e)}$$

$$\frac{dE_dS^*}{dt} = a_2 \cdot E_d \cdot S^* - (d_2 + k_2) \cdot E_dS^* \qquad \text{(S15.f)}$$

and, consequently, the conservation relations are

$$S_T = S + S^* + E_aS + E_dS^* \qquad \text{(S16.a)}$$

$$E_{d_T} = E_d + E_dS^* \qquad \text{(S16.b)}$$

$$E_{a_T} = E_a + E_aS \qquad \text{(S16.c)}$$

We scanned parameter values randomly with log uniform distribution within the intervals defined on Table S1 and following the approach described in the Methods section.

Kinetic rates for the examples included in Figure 3 B-I

For the Zeroth order regime:

$a_1 = 1401.7\, nM \min^{-1}$, $a_2 = 1075.6\, nM \min^{-1}$
$d_1 = 1027.1 min^{-1}$, $d_2 = 942.05 min^{-1}$
$k_1 = 279.24 min^{-1}$, $k_2 = 125.2424 min^{-1}$
$S_T = 97.53\, nM$, $E_{d_T} = 10.12\, nM$
$K_a = 0.0096$, $K_d = 0.0102$

For the First order regime:

$a_1 = 39.44\ nM\min^{-1}$, $a_2 = 30.12\ nM\min^{-1}$
$d_1 = 1280.5 min^{-1}$, $d_2 = 1172.9 min^{-1}$
$k_1 = 547.6 min^{-1}$, $k_2 = 344.19 min^{-1}$
$S_T = 0.49\ nM$, $E_{d_T} = 0.78\ nM$
$K_a = 93.21$, $K_d = 101.32$

1b. Michaelis-Menten approximation

Using the Michaelis-Menten approximation and assuming that the total amount of substrate is S_T=S+S* (in this sum we neglected the amount of substrate bound to the activating and deactivating enzymes), the system can be described by the equation:

$$\frac{dy}{dt_n} = \frac{x(t_n)(1-y)}{K_a+(1-y)} - \frac{y}{K_d+y} \qquad \text{(S17)}$$

where x=(k_1 E_a)/(k_2 E_d) is the input to this system, expressed as the ratio of the maximum velocities of the activating and deactivating enzymatic reactions, with catalytic rate constants k_1 and k_2 ; y=S*/S_T is the output, the fraction of active substrate; $K_a = K_{m,a}/S_T$ and $K_d = K_{m,d}/S_T$ are the Michaelis-Menten constants relative to the total amount of substrate; and time is expressed in units of a reference time t_{ref}=S_T/(k_2 E_d), so that t_n=t/t_{ref}. .

As in the full mechanistic description, we assumed that the activating enzyme increases following an exponential function. This function is characterized by a maximum value $E_{a,max}$ and a characteristic time τ_{Ea}, resulting in:

$$x(t_n) = x_{max}\left(1 - exp\left(-t_n/\tau_{E\,a,n}\right)\right) \qquad \text{(S18)}$$

where $x_{max} = \frac{k_1\,E_{a,max}}{k_2\,E_d}$ and $\tau_{\,E_a,n} = \tau_{E\,a}/t_{ref}$.

Notably, simulation results in the four situations considered (first- or zero-order, fast or slow input) (Fig. S1) are in complete agreement with the results obtained with the full mechanistic description presented in the main text:

1) the input-output curve *shifted* from right to left over time;

2) when the stimulus increased slowly, the leftward *shift* in the input-output curve was correspondingly slow;

3) the *shift* is faster in zero-order, indicating that there is less time for PRESS when the enzymes are saturated;

4) while the n_H decreased with time in the first-order regime**,** it increased in the zero-order regime, from about 1 towards its final high steady-state value (~25 in our simulation). Regarding the first-order regime, we noted in the main text that $n_H(t)$ has a fast increase up to 1.42 and then decreases to 1. This initial increase up to 1.42, which does not appear in the Michaelis-Menten version, is probably due to the intermediary steps (complex formation): at short times, the overall rate of output production depends more on complex formation than catalysis itself.

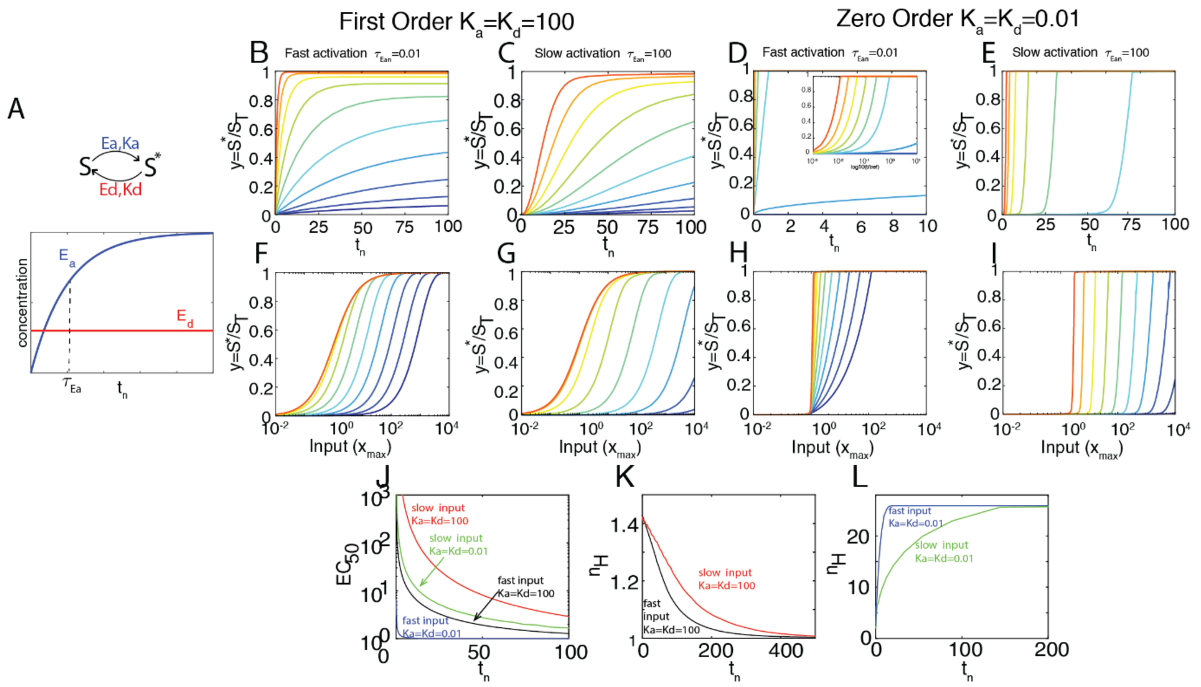

Figure S1: Covalent modification (CM) cycles, with a Michaelis-Menten description, are shifters. A. Top. Diagram of the enzymatic cycle. Bottom. Plot of the dynamics of E_a and E_d for exponential accumulation of E_a. **B-E**. Time courses of y curves for different values of input, x_{max} (in a heatmap scale). **F-I.** Input-output curves at different times (in a heatmap scale). K_a=K_d=100 indicates that the CM cycle is in first order, while K_a=K_d=0.01, indicates it is in zeroth order. $\tau_{Ean} = 0.01$ indicates the input rises fast while $\tau_{Ean} = 100$ indicates it rises slowly. In **B-E**, x_{max} goes from 0.1 to 100 (0.1, 0.21, 0.46, 1, 2.15, 4.64, 10, 21.54, 46.42, 100); in **F and G**, x_{max} goes from 0.1 to 1000 (0.1, 0.28, 0.77, 2.15, 6, 16.68, 46.42, 129.15, 359.38, 1000); in **H,** x_{max} goes from 0.1 to 10 (0.1, 0.16, 0.27, 0.46, 0.77, 1.29, 2.15, 3.59, 5.99, 10); and in **I** x_{max} goes from 1 to 100 (1, 1.66, 2.78, 4.64, 7.74, 12.91, 21.54, 35.93, 59.94, 100). **J-L.** Time courses of EC_{50} (**J**) or n_H (**K** and **L**) for fast ($\tau_{Ea,n}$ = 0.01) or slow ($\tau_{Ea,n}$ = 100) rising input, in the first-order (K_a=K_d=100) or zeroth-order (K_a=K_d=0.01) regimes.

3. Composing Shifters: A ligand-receptor activates a covalent modification cycle.

The full set of reactions for this model is composed of those for a ligand receptor (LR) system and those for a CM cycle, with an extra reaction involving the binding of the receptor and the substrate without the ligand (S19b). This last reaction does not lead to product formation. The reactions are as follows:

$$L + R \underset{k_{off}}{\overset{k_{on}}{\rightleftarrows}} RL \tag{S19.a}$$

$$S + R \underset{d_1}{\overset{a_1}{\rightleftarrows}} RS \tag{S19.b}$$

$$L + RS \underset{k_{off}}{\overset{k_{on}}{\rightleftarrows}} RLS \overset{k_1}{\longrightarrow} S^* + RL \tag{S19.c}$$

$$S + RL \underset{d_1}{\overset{a_1}{\rightleftarrows}} RLS \overset{k_1}{\longrightarrow} S^* + RL \tag{S19.d}$$

$$S^* + E_D \underset{d_2}{\overset{a_2}{\rightleftarrows}} ES^* \overset{k_2}{\longrightarrow} S + E_D \tag{S19.e}$$

For step stimulation, we described the system with the following differential equations:

$$\frac{dR}{dt} = -k_{off} \cdot L/K_D \cdot R + k_{off} \cdot RL - S \cdot R \cdot a_1 + RS \cdot d_1 \tag{S20.a}$$

$$\frac{dRL}{dt} = k_{off} \cdot L/K_D \cdot R - k_{off} \cdot RL - a_1 \cdot S \cdot RL + (k_1 + d_1) \cdot RLS \tag{S20.b}$$

$$\frac{dS}{dt} = d_1 \cdot RLS + k_2 \cdot ES^* - a_1 \cdot RL \cdot S - S \cdot R \cdot a_1 + RS \cdot d_1 \tag{S20.c}$$

$$\frac{dS^*}{dt} = k_1 \cdot RLS + d_2 \cdot S^*E - a_2 \cdot E \cdot S^* \tag{S20.d}$$

$$\frac{dRS}{dt} = -L/K_D \cdot k_{off} \cdot RS + RLS \cdot k_{off} - RS \cdot d_1 + S \cdot R \cdot a_1 \tag{S20.e}$$

$$\frac{dRLS}{dt} = -RLS \cdot k_{off} + RS \cdot L/K_D \cdot k_{off} + a_1 \cdot S \cdot RL - (k_1 + d_1)RLS \tag{S20.f}$$

$$\frac{dS^*E}{dt} = a_2 \cdot S^* \cdot E - (k_2 + d_2) \cdot ES^* \tag{S20.g}$$

$$\frac{dE_d}{dt} = (d_2 + k_2) \cdot ES^* - a_2 \cdot S^* \cdot E, \tag{S20.h}$$

together with the conservation relations

$$R_T = R + RS + RL + RLS \qquad \text{(S21.a)}$$
$$E_T = E_D + ES^* \qquad \text{(S21.b)}$$
$$S_T = S + S^* + RLS + ES^* + RS \qquad \text{(S21.c)}$$

where $K_D = k_{off}/k_{on}$ is the dissociation constant for the ligand-receptor reaction, R_T is the total amount of receptor, k_{off} is the unbinding rate for the ligand-receptor reaction, RS is the inactive receptor-substrate complex, RL is the ligand-receptor complex and the active enzyme for the CM cycle, S_T the total amount of substrate, E_d is the amount of free deactivating enzyme. S^* is the substrate in its active form and S the substrate in its inactive form. The substrate S binds the receptor in either of its two forms: free (R) or bounded to the ligand (RL). Binding and unbinding rates for the substrate to R and RL are a_1 and d_1, respectively. We assume that R binds L and S independently. Hence, the kinetic rates are identical. Finally, S* is produced and the enzyme (RLS) released, with rate k_1. Similarly, S^* binds reversible to its deactivating enzyme E_d with rates a_2 and d_2, forming the intermediate complex ES^*, and releasing S and E_d with rate k_2. Time is dimensionless, $t_n = t/t_{ref}$ with $t_{ref} = S_T/\ (k_1\ E_d)$ being a reference time. We define, as in the main text, the dimensionless Michaelis-Menten parameters $K_a = K_{m,a}/S_T$, and $K_d = K_{m,d}/S_T$, being $K_{m,a}$ and $K_{m,d}$ the dimensional values of the Michaelis-Menten constants.

4. Cascade of covalent modification cycles

The mechanistic model of a cascade of CM cycles is based on a three-tier mitogen activated protein kinase cascade, captured by the following reactions:

$$KKK + E_1 \underset{d_1}{\overset{a_1}{\rightleftarrows}} KKK^* \cdot E_1 \overset{k_1}{\longrightarrow} KKK^* + E_1 \quad \text{(S22.a)}$$

$$KKK^* + E_2 \underset{d_2}{\overset{a_2}{\rightleftarrows}} KKK^* \cdot E_2 \overset{k_2}{\longrightarrow} KKK + E_2 \quad \text{(S22.b)}$$

$$KK + KKK^* \underset{d_3}{\overset{a_3}{\rightleftarrows}} KK \cdot KKK^* \overset{k_3}{\longrightarrow} KP + KKK^* \quad \text{(S22.c)}$$

$$KKP + KKP'ase \underset{d_4}{\overset{a_4}{\rightleftarrows}} KKP \cdot KKP'ase \overset{k_4}{\longrightarrow} KK + KKP'ase \quad \text{(S22.d)}$$

$$KKP + KKK^* \underset{d_5}{\overset{a_5}{\rightleftarrows}} KKP \cdot KKK^* \overset{k_5}{\longrightarrow} KKPP + KKK^* \quad \text{(S22.e)}$$

$$KKPP + KKP'ase \underset{d_6}{\overset{a_6}{\rightleftarrows}} KKPP \cdot KKP'ase \overset{k_6}{\longrightarrow} KKP + KKP'ase \quad \text{(S22.f)}$$

$$KKPP + K \underset{d_7}{\overset{a_7}{\rightleftarrows}} KKPP \cdot K \overset{k_7}{\longrightarrow} KKPP + KP \quad \text{(S22.g)}$$

$$KP + KP'ase \underset{d_8}{\overset{a_8}{\rightleftarrows}} KP \cdot KKP'ase \overset{k_8}{\longrightarrow} K + KP'ase \quad \text{(S22.h)}$$

$$KP + KKPP \underset{d_9}{\overset{a_9}{\rightleftarrows}} KP \cdot KKPP \overset{k_9}{\longrightarrow} KPP + KKPP \quad \text{(S22.i)}$$

$$KPP + KP'ase \underset{d_{10}}{\overset{a_{10}}{\rightleftarrows}} KKPP \cdot KKP'ase \overset{k_{10}}{\longrightarrow} KP + KP'ase \quad \text{(S22.j)}$$

To simplify the notation, we avoided MAP in each variable ($MAPKK$ is named KK, and so on). Each of the three kinases has a dedicated phosphatase, termed after its substrate kinase with the suffix "P'ase". In this model, the reactants in abundance (ATP, water, and so) are assumed to be constant, so they are included in the rate constants.

The 10 reactions described above give rise to 18 differential equations:

$$\frac{d[KKK]}{dt} = -a_1[KKK][E_1] + d_1[KKK \cdot E_1] + k_2[KKK^* \cdot E_2] \quad \text{(S23.a)}$$

$$\frac{d[KKK \cdot E_1]}{dt} = a_1[KKK][E_1] - (d_1 + k_1)[KKK \cdot E_1] \quad \text{(S23.b)}$$

$$\frac{d[KKK^*]}{dt} = -a_2[KKK^*][E_2] + d_2[KKK^* \cdot E_2] + k_1[KKK \cdot \; E_1] + (k_3 + d_3)[KK \cdot KKK^*] - a_3[KKK^*][KK] + (k_5 + d_5)[KKP \cdot KKK^*] - a_5[KKP][KKK^*]$$

(S5S23.c)

$\frac{d[KKK^*\cdot E_2]}{dt} = a_2[KKK^*] - (d_2 + k_2)[KKK^* \cdot E_2]$ (S23.d)

$\frac{d[KK]}{dt} = -a_3[KK][KKK^*] + d_3[KK \cdot KKK^*] + k_4[KKP \cdot KKP'ase]$ (S23.e)

$\frac{d[KK\cdot KKK^*]}{dt} = a_3[KK][KKK^*] - (d_3 + k_3)[KK \cdot KKK^*]$ (S23.f)

$\frac{d[KKP]}{dt} = -a_4[KKP][KKP'ase] + d_4[KKP \cdot KKP'ase] + k_3[KKK \cdot KKK^*] + k_6[KKPP \cdot KKP'ase] + d_5[KKP \cdot KKK^*] - a_5[KKP][KKP'ase]$ (S23.g)

$\frac{d[KKP\cdot KKP'ase]}{dt} = a_4[KKP][KKP'ase] - (d_4 + k_4)[KKP \cdot KKP'ase]$ (S23.h)

$\frac{d[KKP\cdot KKK^*]}{dt} = a_5[KKP][KKK^*] - (d_5 + k_5)[KKP \cdot KKK^*]$ (S23.i)

$\frac{d[KKPP]}{dt} = k_5[KKP \cdot KKK^*] - a_6[KKPP][KKP'ase] + d_6[KKPP \cdot KKP'ase] - a_7[KKPP][K] + (d_7 + k_7)[K \cdot KKPP] + (d_9 + k_9)[KP \cdot KKPP] - a_9[KP][KKPP]$ (S23.j)

$\frac{d[KKPP\cdot KKP'ase]}{dt} = a_6[KKPP][KKP'ase] - (d_6 + k_6)[KKPP \cdot KKP'ase]$ (S23.k)

$\frac{dK}{dt} = -a_7[K][KKPP] + d_7[KK \cdot KKPP] + k_8[KP \cdot KP'ase]$ (S23.l)

$\frac{d[K\cdot KKPP]}{dt} = a_7[K][KKPP] - (d_7 + k_7)[K \cdot KKPP]$ (S23.m)

$\frac{d[KP]}{dt} = \mathrm{k_7[K\cdot\ KKPP]} - \mathrm{a_8}[KP][KP'ase] + d_8[KP \cdot KP'ase] - a_9[KP][KKPP] + d_9[KP \cdot KKPP] + k_{10}[KPP \cdot KP'ase]$ (S23.n)

$\frac{d[KP\cdot KP'ase]}{dt} = a_8[KP][KP'ase] - (d_8 + k_8)[KP \cdot KP'ase]$ (S23.o)

$\frac{d[KP\cdot KKPP]}{dt} = a_9[KP][KKPP] - (d_9 + k_9)[KP \cdot KKPP]$ (S23.p)

$\frac{d[KPP]}{dt} = -a_{10}[KPP][KP'ase] + d_{10}[KPP \cdot KP'ase] + k_9[KP \cdot KKPP]$ (S23.q)

$\frac{d[KKP\cdot KP'ase]}{dt} = a_{10}[KPP][KP'ase] - (d_{10} + k_{10})[KPP \cdot KP'ase]$ (S23.r)

The square brackets indicate concentration of each species. In addition, there are seven conservation equations

$[KKK_{tot}] = [KKK] + [KKK^*] + [KKK \cdot E_1] + [KKK^* \cdot E_2] + [KKK^* \cdot K] + [KKK^* \cdot KP]$ (S24.a)

$[E_{1tot}] = [E_1] + [KKK \cdot E_1]$ (S24.a)

$[E_{2tot}] = [E_2] + [KKK^* \cdot E_2]$ (S24.b)

$[KK_{tot}] = [KK] + [KKP] + [KKPP] + [KK \cdot KKK^*] + [KKP \cdot KKK^*] + [KKP \cdot KKP'ase] + [KKPP \cdot KKP'ase] + [KKPP \cdot K] + [KKPP \cdot KP]$ (S24.c)

$[KKP'ase_{tot}] = [KKP'ase] + [KKP'ase \cdot KKP] + [KKP'ase \cdot KKPP]$ (S24.d)

$[K_{tot}] = [K] + [KP] + [KPP] + [KKPP \cdot K] + [KKPP \cdot KP] + [KP \cdot KP'ase] + [KPP \cdot KP'ase]$ (S24.e)

$[KP'ase_{tot}] = [KP'ase] + [KP \cdot KP'ase] + [KPP \cdot KP'ase]$ (S24.f)

Based on this system of equations, we computed Input-Output curves for the parameters listed on **Table SI 1**

Parameter	Value
V_{max}	$150\ min^{-1}$
K_m	0.3 μM
r	4
$[KKK_{tot}]$	3 nM
$[KK_{tot}]$,$[K_{tot}]$	1.2 μ M
$[KP'ase_{tot}]$	120 nM
$[KKP'ase_{tot}]$, $[E_{2tot}]$	3nM

Table SI 1: Parameter values used in the simulation, the values were taken from Huang and Ferrell 1996 [1]

The reaction rates are only given as K_m and V_{max} in the literature. Thus, we estimated a_n (enzyme binds substrate), d_n(enzyme releases substrate without modifying it) and k_n (enzyme modifies and releases the substrate) according to the Michaelis-Menten formula and the additional assumption, that d_n and k_n have a constant ratio $r_n = k_n/d_n$. Then the rate constants can be calculated by: $d_n = V_{max,n}$, $a_n = \frac{V_{max}}{K_m}(1 + r)$, $k_n = rV_{max,n}$. As proposed by[5], we choose $r = 4$.

We scanned 100 values of the input E_1 from $10^{-3}Nm$ for $1\mu M$ in logarithmic scale for different times, the output was the concentration of the doubly phosphorylated MAPK,$[KPP]$. We computed Input-Output curves using the ode23s function from Matlab to integrate.

5. Transcriptional regulation

Time window and maximal EC_{50} correlation

Analyzing the values of TW and ME for each parameter set, we found that they have a high correlation in the log-log space, $corr\left(log10(ME), log10(TW)\right) = \rho = 0.8562$.

In the main text we showed the results for the Maximal EC_{50} for all combinations of parameter pairs. Due to the high correlation just described, the heatmaps for the Time Window are qualitatively similar, and leads to similar conclusions (Figure S2).

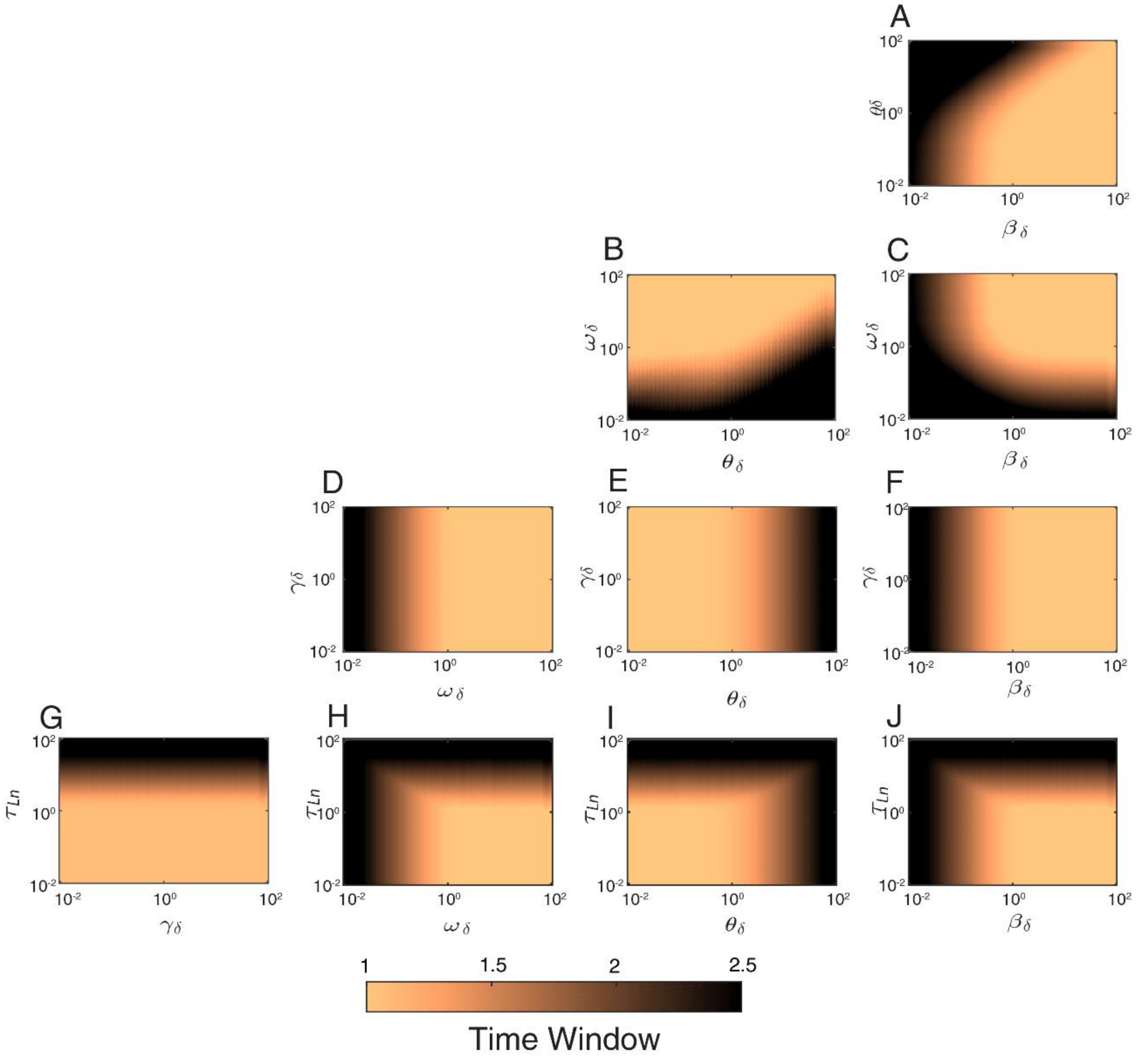

Figure S2: Time window in color scale for all different combination of parameter pairs.

References

1. Huang, C.Y. and J.E. Ferrell, Jr., *Ultrasensitivity in the mitogen-activated protein kinase cascade.* Proc Natl Acad Sci U S A, 1996. **93**(19): p. 10078-83.
2. Bhalla, U.S. and R. Iyengar, *Emergent properties of networks of biological signaling pathways.* Science, 1999. **283**(5400): p. 381-7.
3. Ventura, A.C., et al., *Utilization of extracellular information before ligand-receptor binding reaches equilibrium expands and shifts the input dynamic range.* Proc Natl Acad Sci U S A, 2014. **111**(37): p. E3860-9.
4. Ossareh, H.R., et al., *Long signaling cascades tend to attenuate retroactivity.* Biophys J, 2011. **100**(7): p. 1617-26.
5. Bluthgen, N. and H. Herzel, *How robust are switches in intracellular signaling cascades?* J Theor Biol, 2003. **225**(3): p. 293-300.
